# Expression and Complex Formation of MMP9, MMP2, NGAL, and TIMP1 in Porcine Myocardium but Not in Skeletal Muscles in Male Pigs with Tachycardia-Induced Systolic Heart Failure

**DOI:** 10.1155/2013/283856

**Published:** 2013-04-22

**Authors:** Liliana Kiczak, Alicja Tomaszek, Jacek Bania, Urszula Paslawska, Maciej Zacharski, Agnieszka Noszczyk-Nowak, Adrian Janiszewski, Piotr Skrzypczak, Hossein Ardehali, Ewa A. Jankowska, Piotr Ponikowski

**Affiliations:** ^1^Regional Specialist Hospital in Wroclaw, Research and Development Centre, Wroclaw, Poland; ^2^Department of Biochemistry, Pharmacology and Toxicology, Faculty of Veterinary Medicine, Wroclaw University of Environmental and Life Sciences, ul. C.K. Norwida 31, 50-375 Wroclaw, Poland; ^3^Department of Food Hygiene and Consumer Health Protection, Faculty of Veterinary Medicine, Wroclaw University of Environmental and Life Sciences, Wroclaw, Poland; ^4^Department of Internal and Diseases with Clinic for Horses, Dogs and Cats, Faculty of Veterinary Medicine, Wroclaw University of Environmental and Life Sciences, Wroclaw, Poland; ^5^Department and Clinic of Surgery, Faculty of Veterinary Medicine, Wroclaw University of Environmental and Life Sciences, Wroclaw, Poland; ^6^Department of Heart Diseases, Wroclaw Medical University, Wroclaw, Poland; ^7^Division of Cardiology, Department of Medicine, Northwestern University School of Medicine, Chicago, USA

## Abstract

Matrix metalloproteinases (MMPs) are involved in the remodeling of extracellular matrix in various tissues. Their functioning could be related to the formation of complexes, containing MMP9, MMP2, tissue inhibitor of metalloproteinases type 1 (TIMP1), and neutrophil gelatinase-associated lipocalin (NGAL). Such complexes have not been investigated in either myocardial or skeletal muscles. We examined 20 male pigs with heart failure (HF), and 5 sham-operated animals. There were no differences in the mRNA expression of MMP9, MMP2, TIMP1, and NGAL between diseased and healthy animals, in either left ventricle (LV) myocardium or skeletal muscles. In LV from both diseased and healthy animals, in nonreducing and nondenaturing conditions, we demonstrated the presence of high molecular weight (HMW) complexes (130, 170, and 220 kDa) containing MMP9, TIMP1, and NGAL (also MMP2 in 220 kDa complex) without proteolytic activity, and a proteolytically active 115 kDa MMP9 form together with 72 and 68 kDa bands (proMMP2 and MMP2). Proteolytically active bands were also spontaneously released from HMW complexes. In skeletal muscles from both diseased and healthy animals, in nonreducing and nondenaturing conditions, we found no HMW complexes, and proteolytic activity was associated with the presence of 72 and 68 kDa bands (proMMP2 and MMP2).

## 1. Introduction

Matrix metalloproteinases (MMPs) are the key enzymes orchestrating the turnover of extracellular matrix (ECM) of virtually all tissues [1]. MMP2 and MMP9 are considered to play a pivotal role in myocardial remodeling in a number of cardiovascular diseases, including myocardial infarction (MI) and ischemic and idiopathic dilated cardiomyopathy (DCM) [2–4]. There are premises that they could also be involved in structural and functional changes in skeletal muscles contributing to the development of skeletal myopathy [5], which constitutes an important pathophysiological element aggravating the progression of heart failure (HF) [6].

MMP9 and MMP2 are released as proenzymes (pro-MMPs) into extracellular space, where they are activated through an enzymatic cleavage to mature MMPs [4]. The proteolytic activity of MMPs is critical for the ongoing processes of tissue remodeling and repair [7], and therefore, it is tightly controlled through different mechanisms including interaction with inhibitors (tissue inhibitors of metalloproteinases, TIMP1-4) or stabilizers of active enzymes (neutrophil gelatinase-associated lipocalin, NGAL) [8]. Both pro-MMPs and MMPs can form the noncovalent high molecular weight (HMW) complexes, which may contain different molecules, such as TIMPs [7] or/and NGAL [9, 10]. There are premises that the stability and the proteolytic activity of MMPs forming such complexes vary from the properties of monomeric forms [8]. HMW complex composed of MMP9, MMP2, MMP3, TIMP1, and TIMP2 found in rat chorioallantoid membranes has been shown to be stabile and latent at high physiological calcium concentrations, dissociating at low calcium concentration and releasing active enzymes [11]. 

There is also available evidence suggesting that the MMP complexes are present and active in biological fluids. Complexes containing proMMP9, NGAL, and TIMP1 have been demonstrated in the medium from cultured human neutrophils [12], MMP9/NGAL complexes—in the synovial fluid of patients with osteoarthritis [13], MMP9/TIMP1 complexes—in the urine of patients with prostate and bladder carcinoma [14], whereas MMP9/NGAL complexes—in the medium from human cholangiocarcinoma cell cultures [15].

Although the presence and proteolytic activity of MMP9 and MMP2 have been confirmed in both normal and failing hearts [4] as well as in skeletal muscles [5], the formation of HMW complexes containing these 2 major proteases and associated enzymatic activity has not been comprehensively studied in these tissues. Due to the obvious difficulties in obtaining serial human tissue samples, we used porcine model of pacing-induced (nonischaemic) dilated cardiomyopathy (DCM) [16]. Our model is based on a relatively slow, long-term right ventricular (RV) pacing, which is able to induce myocardial dysfunction confirmed by both echocardiography and molecular methods, accompanied by an occurrence of clinical features of the HF syndrome [16]. In this paper, we investigated the mRNA and protein expression of MMP9, MMP2, NGAL and TIMP1, along with the levels of complexes and their proteolytic activity in left ventricle (LV) myocardium and skeletal muscles (*biceps femoris*, BF) from male pigs with and without RV pacing-induced chronic systolic HF.

## 2. Methods

### 2.1. Protocol of the Development of Right Ventricle Pacing-Induced Cardiomyopathy

The study was performed in 25 adult pigs of Polish Large White breed (sibling 8-month-old males, initially weighing from 115 to 125 kg). All animals were treated and cared for in compliance with the Guide for the Care and Use of Laboratory Animals as published by the National Institutes of Health (NIH publication No. 85-23, revised in 1996). All experiments were performed in compliance with the Bioethical Committee of the Wroclaw University of Environmental and Life Sciences guidelines for the experimentation on animals.

We developed the experimental porcine model of chronic systolic nonischaemic HF, that is, tachycardia-induced cardiomyopathy (TIC), which was previously established by our group in female animals [16]. Briefly, single-chamber pacemakers (SENSIA SESR01, Medtronic, Poland) were implanted in all 25 pigs, with a bipolar screw-in pacing transvenous lead (CAPSUREFIX NOVUS 58 cm, Medtronic, Poland) positioned at the apex of RV. The pacemakers were programmed for sequential RV pacing at 170 bpm in 20 randomly chosen animals, whereas 5 remaining pigs served as sham-operated controls.

### 2.2. Clinical Assessment and Classification of Pigs to Subsequent Stages of HF

All animals remained under the same everyday clinical care. There was no difference in the measurement protocols between the paced and nonpaced male pigs. The assessments were performed regularly on a monthly basis and comprised (a) clinical assessments including the recording of HF signs and symptoms (for details see below) and (b) transthoracic echocardiography (for details see below).

The following signs and symptoms of HF occurring in examined male pigs were evaluated semiquantified using a 0–3 scale (0—no sign/symptom, 3—very severe intensity of a particular sign/symptom): appetite, interest in surroundings, willingness to undertake physical activity (after forcing), dyspnea after exertion, lying down after exertion (fatigue), dyspnea at rest, ascites, redness of snouts and ears after exertion, and snout and ears cyanosis at rest. All scores were averaged for each pig for the particular time point. The following ranges of averaged scores were used for the categorization of pigs to subsequent clinical stages of HF: mild HF (≥0 and ≤1), moderate HF (>1 and ≤2), and severe HF (>2 and ≤3). It was prospectively designed that animals developing the consecutive stages of HF (mild, moderate, and severe) during the experiment would be presented for euthanasia. Control animals underwent euthanasia parallel to TIC pigs and were selected for this procedure in a random manner.

All pigs were euthanized with an overdose of pentobarbital, and the postmortem examinations were performed. Tissue sections from left ventricle (LV) myocardial free wall and skeletal muscles (biceps femoris muscle, BF) were taken and immediately frozen in liquid nitrogen before the further molecular analyses were performed.

### 2.3. Transthoracic Echocardiography

Transthoracic echocardiography was performed using an imaging ultrasound system (Aloka 4000+ with a 3.5 MHz phased array transducer). Right parasternal but not left apical views were readily visible in all animals. Two-dimension and direct M-mode echocardiography was performed at the right parasternal area in left lateral decubitus position. M-mode tracing was carried out from a long four-chamber view, just below the mitral valve. Diastolic measurements were performed at the onset of the QRS complex of ECG. Systolic measurements were performed at the end of T wave. The ratio of left atrium and aorta diameters (LA/Ao) was measured in diastole in a 2D short axis at the level of aortic valve. LV end-diastolic diameter (LVEDd, cm) and LV end-systolic diameter (LVESd, cm) were measured using the leading-edge method from at least 3 consecutive cardiac cycles as recommended by the American Society for Echocardiography [17]. LV fractional shortening (LVFS) was calculated as a ratio of (LVEDd-LVESd) and LVESd and expressed as a percentage. Using the Teicholz formula [18], LV end-diastolic volume (LVEDV, mL) and LV end-systolic volume (LVESV, mL) LV ejection fraction (LVEF, %), and stroke volume (SV, mL) were computed. LVPW (LV posterior wall) thickening was calculated using the equation   = [(LVPWs − LVPWd/LVPWd)] × 100 and expressed as a percentage (LVPWs—at systole, LVPWd—at diastole).

Tissue Doppler imaging was performed from the right parasternal short axis view. Doppler cursor was placed at the LV free wall below the mitral annulus at the level of papillary muscle. The recorded velocities (m/s) represented the contraction of circumferential layer of myocardium, that is, negative diastolic waves Em (early diastolic) and Am (late diastolic) waves. Tracings were recorded at a sweep speed of 100 mm/s and measurements were averaged for 3 separate heart beats.

### 2.4. Neurohormonal Activation

Venous blood samples were drawn from each animal directly before euthanasia and immediately centrifuged, followed by processing and storage as serum and plasma samples at −80°C until further analyses. Plasma renin activity (PRA) was assayed using an indirect radioimmunoassay (REN-CT2, CIS Bio International, POLATOM, Poland) according to the manufacturer's instructions. Values were expressed as ng/mL/h of a generated angiotensin I. Serum B-type natriuretic peptide (BNP, ng/mL) was assessed at 1 : 5 dilution using a Peptide enzyme immunoassay (EIA) Kits (Bachem, St. Helens, UK) according to the manufacturer's instructions. PRA and BNP measurements were performed in duplicates.

### 2.5. Quantitative RT-PCR

Total RNA was prepared from 30 mg samples of porcine LV myocardium and BF tissues using the RNeasy Fibrous Tissue Mini Kit (Qiagen, Poland) according to the manufacturer's instructions. The protocol included an on-column DNase digestion to remove the genomic DNA. First-strand cDNA was synthesized using a SuperScript III First-Strand Synthesis System with oligo(dT)_20_ primer (Invitrogen, Poland).

Based on the genomic and cDNA sequences, the primers for MMP9, MMP2, TIMP1, NGAL, and GAPDH (glyceraldehyde-3-phosphate dehydrogenase) were designed using the Molecular Beacon Software (Bio-Rad) (Table 1). The primers spanned exon junctions to prevent the amplification of genomic DNA. The GAPDH gene was chosen as a reference to normalize the differences in the amount of RNA and in the efficiency of reverse transcription.

The relative amounts of porcine MMP9, MMP2, TIMP1, and NGAL in LV myocardium and BF samples were determined using the quantitative real-time PCR using the iQ5 optical system (Bio-Rad) with the kapa mix (Kapa Biosystems, MA, USA) as appropriate. The reactions were performed under the following conditions: an initial denaturation of 94°C for 10 min, 35 cycles of 94°C for 30 s, 65°C for 30 s, followed by 72°C for 1 min. All measurements were performed in triplicates. The specificity of PCR was determined using a melt-curve analysis for each reaction. PCR products for all subsequent investigated genes were sequenced (Genomed, Poland) to confirm their identity.

The amplification efficiency was established by running a template at successive dilutions. Successive dilutions were plotted against the appropriate CT values to generate a standard curve. The slope calculated from the standard curve was used to determine the amplification efficiency (*E*) according to the formula: *E* = 10^1/slope^. Since the amplification efficiencies for the target amplicons and GAPDH were not comparable, the Pfaffl method was used to determine the relative expression [19]. mRNA expression was presented in arbitrary units (AU), where the sample from LV myocardium (or BF) from one of the control pigs was chosen as the calibrator, and its mRNA expression was considered as 1.

### 2.6. Immunoblotting

LV myocardium and BF samples (30 mg of each) were homogenized in 200 *μ*L of an ice-cold extraction buffer (50 mM Tris-HCl, 200 mM NaCl, 10 mM CaCl_2_, 1% Triton X-100, pH 7.6) [20] containing a 1 : 50 protease inhibitor cocktail (Sigma-Aldrich, Poland). After incubation on ice (30 min) and centrifugation at 9700 ×g, the supernatants were collected and stored on ice. Next, insoluble material was extracted twice after a 10 min incubation with 50 *μ*L of an ice-cold extraction buffer, and supernatants from all extractions were combined. Protein quantification was performed using the Bradford reagent (Sigma-Aldrich), according to the manufacturer's instructions.

Protein samples (0.5–25.0 *μ*g) were mixed with a nonreducing sample buffer (63 mM Tris, 10% glycerol, 2% SDS, 0.1% bromophenol blue, pH 6.8), followed by incubation for 5 min at a room temperature (nonreducing, nondenaturing conditions). Protein samples (25 *μ*g) were also prepared in a reducing sample buffer (Pierce, Poland) (with a final dithiothreitol (DTT) concentration of 0.1 M) followed by incubation for 5 minutes at either a room temperature (reducing and nondenaturing conditions) or 95°C (reducing and denaturing conditions).

All samples were subsequently separated on a 10% gel in a SDS-PAGE and transferred onto the PDVF membrane (Millipore, Poland). The membrane was treated with the Quentix signal enhancer (Pierce), blocked for 1 h with 5% nonfat milk in the PBS containing 0.5% (v/v) Triton X-100 (Sigma-Aldrich), and incubated overnight with polyclonal goat antibodies against MMP9, TIMP1, or NGAL (1 : 500) (R&D System, Poland) or with monoclonal mouse anti-MMP2 antibodies (1 : 1000) (Millipore). Blots were developed using the SuperSignal West Femto ECL substrate (Pierce). Recombinant proteins, NGAL (R&D System, Poland), TIMP1 (R&D System, Poland), proMMP2 (Sigma-Aldrich, Poland) and the culture media from DH82 cell line (MMP9 source) prepared in nonreducing, nondenaturing, as well as in reducing, nondenaturing, and reducing, and denaturing conditions, were used as positive controls. 

### 2.7. Coimmunoprecipitation

For the procedure of coimmunoprecipitation of HMW complexes containing MMP9, homogenates from LV myocardium (suspended in an extraction buffer) were initially incubated with protein G agarose (Sigma-Aldrich). The supernatant was mixed with 25 *μ*L of protein G agarose and anti-MMP9 antibodies (the same antibodies which were used for Western blotting) (5*μ*g). After a 60 min incubation, the protein G agarose beads were washed extensively and proteins were eluted with 50 *μ*L of an elution buffer (pH 2.8, Pierce), neutralized with 5 *μ*L of 1 M Tris (pH 9.0), and electrophoresed on a 10% SDS-PAGE (nonreducing and nondenaturing conditions), followed by immunoblotting. Samples immunoprecipitated with anti-MMP9 antibody were blotted using anti-TIMP1, anti-NGAL, and anti-MMP2 antibodies. In all cases, blotting was performed as described in Section 2.6.

### 2.8. Gelatin Zymography

For the detection of MMP2 and MMP9 proteolytic activity in porcine LV homogenates, the classic method, that is, gelatin zymography, was used. In this method, enzyme activity is visible as clear zones in the gelatin-containing gel, where the substrate (gelatin) is digested by enzymes having the gelatinase activity [21]. MMP2 and MMP9 have been shown to specifically digest gelatin [21].

LV myocardium and BF samples were prepared as described in Section 2.6. Protein samples (50 *μ*g) were mixed with a nonreducing sample buffer (63 mM Tris, 10% glycerol, 2% SDS, 0.1% bromophenol blue, pH 6.8) and separated at 4°C on a substrate SDS-PAGE (10% acrylamide, 1 mg/mL gelatin) [20, 22]. The gels were washed 3 times in 2.5% Triton X-100 for 30 min and incubated overnight at 37°C in a collagenase buffer (50 mM Tris-HCl, 200 mM NaCl, 5 mM CaCl_2_, 0.2% Brij-35, and pH 7.6) [23, 24]. Then, the gels were stained using 0.5% Coomassie Blue R-250 (Sigma-Aldrich) in 30% methanol and 10% acetic acid for 60 min and destained in 30% methanol and 10% acetic acid [24]. Gelatinase activity was identified as clear zones against a blue background.

Gelatin zymography of homogenates from porcine LV myocardium was performed twice, that is, first as described above, and second with a modification based on the preincubation of analyzed samples in a collagenase buffer containing 10 mM EDTA (the latter known to inhibit specifically an enzymatic activity of MMPs [25]) in order to prove the specificity of gelatinolytic activity associated with the presence of MMP9 in demonstrated bands. Additionally, the culture media from canine macrophage-like DH82 cell line were used as a positive control of MMP2 and MMP9 activity [26].

### 2.9. Spontaneous Release of Proteolytically Active MMPs

The preparation of homogenates of porcine LV myocardium was described in Section 2.6. 1 mg of extracts of LV myocardium was mixed with the same volume of Brij buffer (0.5 M NaCl, 5 mM CaCI_2_, 50 mM Tris, 0.05% Brij, pH 7.6) [27], 40 *μ*L of gelatin-CNBr-Sepharose4B (Sigma-Aldrich) and incubated at room temperature for 60 min. Due to removing gelatin-Sepharose4B (with attached MMP2 and MMP9), the extract of LV myocardium was depleted of any active MMPs. Subsequently, the extracts of LV myocardium were incubated during 60 min with 25 *μ*L protein G agarose (Sigma-Aldrich). The supernatant was mixed with 25 *μ*L of protein G agarose and antibody against MMP9 (5 *μ*g). After an overnight incubation at 4°C (to prevent the enzyme autodegradation) and the extensive washing, immunoprecipitated proteins were eluted with 50 *μ*L of an elution buffer (pH 2.8, Pierce), neutralized, and incubated for 24 hours at 4°C (to prevent the enzyme autodegradation). Subsequently, the samples were analyzed in gelatin zymography (as described in details in the previous paragraph).

### 2.10. Statistical Analyses

Continuous variables were presented as arithmetic means ± standard errors of a mean (SEM). All molecular and echocardiography assessments were performed in triplicates. Spearman's rank correlatory coefficients were used for all correlatory analyses. *P* < 0.05 was considered as statistically significant. Statistical analyses were performed using the Polish version of Statistica 9.1 (StatSoft, OK, USA).

## 3. Results

### 3.1. Development of Symptomatic Systolic HF in Male Pigs with RV Pacing

In the course of RV pacing, male pigs developed a clinical picture of chronic HF and were presented for euthanasia at subsequent predefined stages of HF: mild (6 ± 2 weeks of pacing, *n* = 7), moderate (11 ± 3 weeks of pacing, *n* = 8), and severe HF (18 ± 4 weeks of pacing, *n* = 5). Sham-operated pigs (*n* = 5) developed no symptoms of HF.

Echocardiography performed directly before euthanasia revealed that RV pacing-induced progressive development of LV systolic and diastolic dysfunction along with LV dilatation with the most marked echocardiography abnormalities seen in pigs with severe symptoms of HF (Table 2). Echocardiography parameters measured in sham-operated pigs remained normal during the entire study (Table 2). Moreover, the development of symptomatic systolic HF was accompanied by the neurohormonal activation, as evidenced by the higher serum levels of BNP and the increased PRA (Table 2).

### 3.2. mRNA Expression of MMP9, MMP2, NGAL, and TIMP1

**Figure 1:**
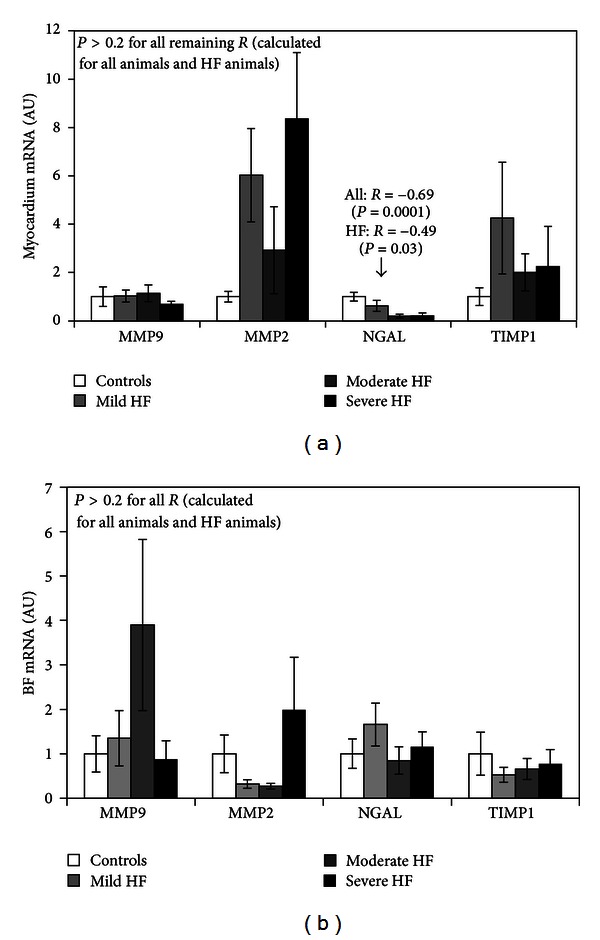
The relative mRNA expression of matrix metalloproteinase type 9 (MMP9), matrix metalloproteinase type 2 (MMP2), neutrophil gelatinase-associated lipocalin (NGAL), and tissue inhibitor of metalloproteinases type 1 (TIMP1) in homogenates from left ventricle (LV) myocardium (a) and biceps femoris (BF) (b) of sham-operated pigs and animals with mild, moderate, and severe HF. Results are expressed in arbitrary units, with a mean value for all control animals considered as 1, separately for each molecule in each tissue. Values are presented as means ± standard error means. *R* stands for the Spearman' rank correlatory coefficient. The relative mRNA expression of MMP9, MMP2, NGAL, and TIMP1 was, respectively, approximately 30, 4, 30, and 10 times lower in porcine homogenates of BF as compared to porcine homogenates of LV myocardium.

There were no differences in the mRNA expression of MMP9, MMP2, and TIMP1 in LV myocardium between diseased and healthy animals (Figure 1(a)). The mRNA expression of NGAL in LV myocardium was approximately 5-fold reduced in pigs with moderate and severe HF as compared to controls (*P* < 0.001) (Figure 1(a)). Also among all examined animals, the reduced mRNA expression of NGAL in LV myocardium was accompanied by LV dilatation (LVEDD: *R* = −0.53, *P* = 0.005), diastolic dysfunction (Em: *R* = 0.52, *P* = 0.005), and increased PRA (*R* = −0.48, *P* = 0.03). Furthermore, there were no differences in the mRNA expression of MMP9, MMP2, NGAL, and TIMP1 in skeletal muscles between diseased and healthy animals (Figure 1(b)). The relative mRNA expression in skeletal muscle was approximately 30-, 4-, 30-, and 10-fold lower than in LV myocardium for MMP9, MMP2, NGAL, and TIMP1, respectively.

### 3.3. Protein Expression of MMP9, MMP2, NGAL, and TIMP1 in LV Myocardium

#### 3.3.1. Nonreducing and nondenaturing Conditions

**Figure 2:**
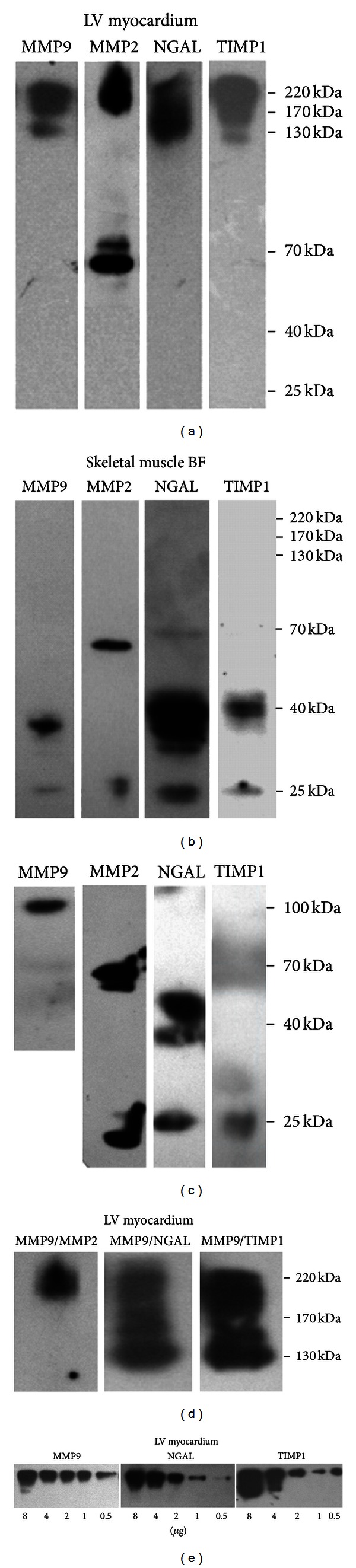
Representative Western blots for MMP9, MMP2, NGAL and TIMP1 detection in porcine homogenates of LV myocardium (a) and BF (b) in nonreducing and nondenaturing conditions. Positive controls in nonreducing and nondenaturing conditions (c). Representative coimmunoprecipitates of porcine lysates of LV myocardium immunoprecipited with anti-MMP9 antibody, and subsequently blotted with anti-MMP2, anti-NGAL, and anti-TIMP1 antibodies (d). Representative semiquantitative Western blots of porcine homogenates of LV myocardium using anti-MMP9, anti-TIMP1, and anti-NGAL antibodies (e).

Blots of LV homogenates with MMP antibody from both diseased and healthy pigs in nonreducing and nondenaturing conditions revealed 3 bands of 130, 170, and 220 kDa. The same bands were seen when anti-NGAL or anti-TIMP1 antibodies were used (Figure 2(a)). However, when LV homogenates were blotted using anti-MMP2 antibody, only a 220 kDa band was detected. Coimmunoprecipitation of LV myocardial tissue using both anti-MMP9 and anti-TIMP1 antibodies, or both anti-MMP9 and anti-NGAL antibodies confirmed that all these 3 bands contained MMP9, NGAL, and TIMP1 (Figure 2(d)). Coimmunoprecipitation of LV myocardial tissue using both anti-MMP9 and anti-MMP2 antibodies revealed that a 220 kDa band contained MMP2 in addition to the other 3 proteins (Figure 2(d)). 

 In order to demonstrate which of the 130, 170, and 220 kDa bands is the most abundant in porcine LV myocardium, Western blotting with either anti-MMP9, anti-TIMP1, or anti-NGAL antibodies with an increasing amount of protein samples (0.5–8 *μ*g) from LV lysates was performed. This experiment demonstrated that the 220-kDa complex was the most abundant in porcine LV myocardium (Figure 2(e)). In LV homogenates, there were no bands corresponding to monomers containing either MMP9, NGAL, or TIMP1, but there were bands of 72 kDa and 68 kDa corresponding to monomeric pro-MMP2 and MMP2, respectively (Figure 2(a)).

#### 3.3.2. Reducing and Nondenaturing Conditions

**Figure 3:**
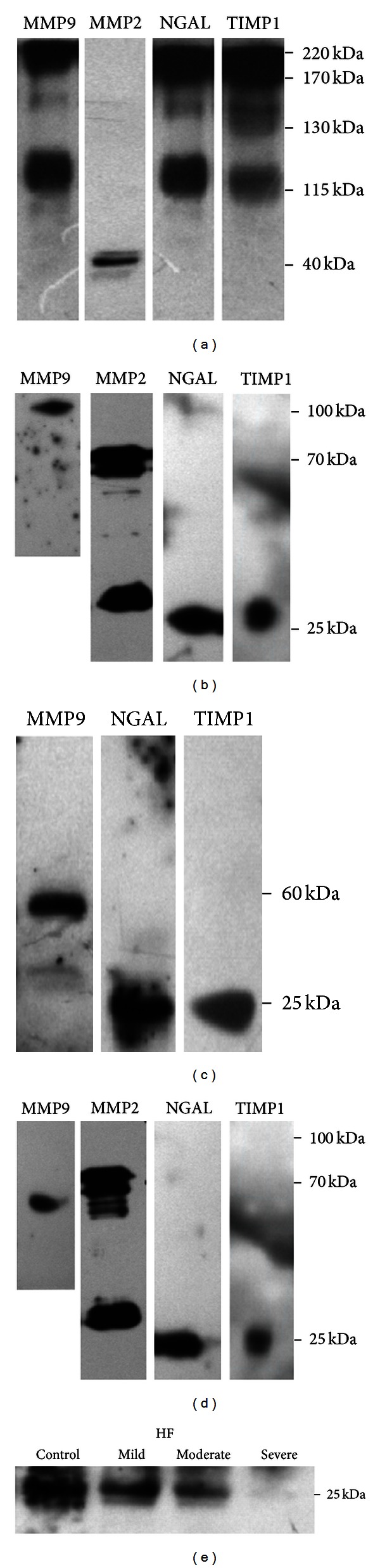
Representative Western blots for MMP9, MMP2, NGAL, and TIMP1 detection in the porcine homogenates of LV myocardium performed in reducing and nondenaturing conditions (a 5-minute incubation in 0.1 M dithiothreitol [DTT] at a room temperature) (a) and in reducing and denaturing conditions (a 5-minute incubation in 0.1 M DTT at 95°C) (c). Positive controls for reducing and nondenaturing conditions (b) and reducing and denaturing conditions (d). Representative Western blots in the porcine homogenates of LV myocardium from a healthy pig and animals with mild, moderate, and severe HF with anti-NGAL antibodies in reducing and denaturing conditions (e).

Western blots on homogenates from LV myocardium performed in reducing conditions (i.e., incubated with DTT) at a room temperature revealed the presence of an intense 220 kDa band containing MMP9, NGAL, and TIMP1 (previously seen in nonreducing conditions), very subtle 130 kDa and 170 kDa bands, along with another intense 115 kDa band containing MMP9, NGAL, and TIMP1. MMP2 was detected in a form of 40-kDa band (Figure 3(a)). The 220 kD complex remained stable in reducing and nondenaturing conditions, suggesting that hydrophobic interactions were involved in the maintenance of its stability. In contrast, the 130 and 170 kDa complexes were destabilized in reducing and nondenaturing conditions, suggesting the involvement of disulphide bonds in their formation. In reducing conditions, there was a 115-kDa band present, which corresponds to active MMP9 band observed in zymography.

#### 3.3.3. Reducing and Denaturing Conditions

Western blots performed with the same samples in both reducing (i.e., incubated with DTT for 5 min at a room temperature) and denaturing conditions (i.e., incubated with DTT for 5 min at 95°C) revealed the presence of a 60 kDa-band (blotted with anti-MMP9 antibody) and a 25 kDa-band (blotted with both anti-NGAL and anti-TIMP1 antibodies), which corresponded to monomers of MMP9, NGAL, and TIMP1 (Figure 3(c)). The optic density of a 25 kDa-band blotted with anti-NGAL antibody was lower in pigs with advanced HF as compared to controls (Figure 3(e)). We did not demonstrate any other bands corresponding to HMW complexes and MMP2 was not detected.

### 3.4. Gelatinase Activity in LV Myocardium

**Figure 4:**
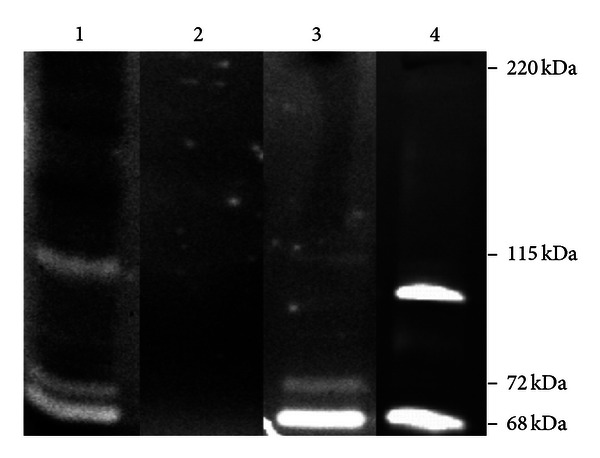
Representative gelatin zymograms of porcine homogenates from LV myocardium with proteolytically active bands of 68, 72, and 115 kDa at baseline (line 1), after the depletion of active gelatinolytic forms using gelatin-Sepharose 4B affinity chromatography (line 2), and subsequently after the 24-hour incubation at 4°C (line 3). Line 4—positive control, the culture media from canine macrophage-like DH82 cell line.

Gelatin zymography of homogenates from LV myocardial tissue from healthy and diseased pigs (performed in nonreducing and nondenaturing conditions) revealed the presence of several active bands ranging from 68 to 115 kDa (Figure 4, line 1). However, the HMW complexes seen in Western blots (130, 170, and 220 kDa) were not detected during gelatin zymography, which confirmed that the proteins of the complex had no enzymatic activity.

### 3.5. Release of Active MMP9 and MMP2 from MMP9/MMP2/NGAL/TIMP1 Complexes Derived from LV Myocardium

Homogenates of LV myocardium (Figure 4(a) line 1) were depleted of active MMP2 and MMP9 species using the gelatin Sepharose affinity chromatography (Figure 4(a) line 2). Presumably inactive complexes were immunoprecipitated using anti-MMP9 antibodies. Immunoprecipitates were incubated for 24 h to enable the potential release of molecules from complexes, while they were kept at 4°C to prevent enzymatic autodegradation. The subsequent gelatinase zymography of these immunoprecipitates demonstrated the presence of 68 kDa, 72 kDa, and 115 kDa bands (Figure 4(a) line 3), suggesting that active forms of the 115-kDa MMP9 and the 68 and 72 kDa MMP2 could be spontaneously released from the HMW complexes.

### 3.6. Protein Expression of MMP9, MMP2, NGAL, and TIMP1 in Skeletal Muscles

Western blots of skeletal muscles from both diseased and healthy pigs (under nonreducing and nondenaturing conditions) and probed with either MMP9, MMP2, TIMP1 or NGAL antibodies revealed the presence of 25 kDa and 40 kDa bands corresponding to monomers and dimers of TIMP1, or/and NGAL. Furthermore, a weak 40 kDa band containing MMP9 and additional 68 and 72 kDa bands containing MMP2 (most likely MMP2 and pro-MMP2, resp.) were also detected. We found no bands of a greater mass corresponding to HMW complexes containing the combination of these 4 molecules (Figure 2(b)).

### 3.7. Gelatinase Activity in Skeletal Muscles

Gelatin zymography of BF homogenates from healthy and diseased pigs (performed in nonreducing nondenaturing conditions) confirmed the presence of only 2 weak bands with gelatinolytic activity of 68 and 72 kDa, corresponding to MMP2 and proMMP2, respectively (data not shown).

## 4. Discussion

In the present study we have demonstrated the presence of HMW complexes containing MMP9, MMP2, TIMP1, and NGAL in LV myocardium (but not in skeletal muscles) of male pigs with and without systolic nonischaemic HF. We have shown that HMW complexes release spontaneously the proteolytically active MMP9 and MMP2.

In this study we have demonstrated that RV pacing at 170 bpm applied in male pigs induces the development of symptomatic systolic HF, which has been evidenced by dilated LV cavity accompanied by impaired systolic and diastolic LV function and neurohormonal activation. Previous porcine TIC models have been developed in young, 20–30 kg weight piglets using rapid pacing at 220–240 bpm up to 3 weeks and resulted in the development of acute or subacute rather than chronic HF [28–31]. Low age of animals used in these studies and the standard castration of male animals for experimental purposes might affect the observations [31]. Taking these facts into consideration, we decided to use adult male pigs initially weighting from 115 to 125 kg and low pacing rate (170 bpm) to develop chronic HF. The experiments on male adult pigs have some limitations, for example, the high costs of maintenance and the aggressive behavior of animals. Our TIC model does not require open-chest surgery, associated with potential risk of intraoperative complications, as in coronary ligation model [32]. While coronary ligation and microembolization create an ischemic HF limited to a specific region of the heart [32], our model could be used to study generalized myocardial dysfunction.

We have demonstrated the presence of both mRNA and protein of MMP9, MMP2, TIMP1, and NGAL in LV myocardium and skeletal muscles of male pigs with and without systolic HF. In fact, there are only few studies reporting the expression of these genes in myocardium [33–35] and skeletal muscles [5] and none of these papers compared the expression of these genes between the 2 aforementioned tissues.

We have found significant differences regarding the mRNA and protein expression of MMP9, MMP2, TIMP1, and NGAL between myocardial and skeletal muscles. Firstly, the mRNA expression of these 4 genes was markedly lower in skeletal muscles as compared to LV myocardium (in both healthy and diseased animals). Secondly, the formation of complexes differed between myocardial and skeletal muscle tissues. In nonreducing and nondenaturing conditions, in LV myocardium there were HMW complexes containing MMP9, MMP2, TIMP1, and NGAL, along with pro-MMP2 and MMP2 monomers, whereas in skeletal muscles only dimers and monomers of these 4 aforementioned molecules were present. Thirdly, in LV myocardium, proteolytic activity was associated with the presence of active MMP2 and MMP9 forms and also the presence of HMW complexes being the reservoir of spontaneously released MMPs, whereas in skeletal muscles the gelatinolytic activity was relatively weak and was associated only with the presence of pro-MMP2 and MMP2 monomers (as compared to LV myocardium). The gelatinolytic activity of skeletal muscles was not extensively studied. Schiøtz Thorud et al. [5] demonstrated the presence of gelatinolytically active MMP2 and MMP9 in skeletal muscles of rats after myocardial infarction and sham-operated controls; however, the amount of mRNA and protein of these MMPs did not differ between healthy and diseased animals. The critical role of the MMPs in physiological and pathophysiological tissue remodeling implies that this complex system needs to be precisely controlled on different levels [8]. Only recently, the formation of complexes including MMPs with different components has received an interest as a potential mechanism with the ability to modify the stability of protease structure and their enzymatic activity [8, 11]. The available evidence on the complexes containing MMPs is rather scarce and most of the studies were mainly performed in biological fluids [13–15] and media of cultured cells and tissues [36, 37]. Rouet-Benzineb et al. [38] demonstrated the colocalization of MMP2 and MMP9 in LV myocardium from patients with idiopathic DCM using a confocal microscopic immunoreactive staining. However, only one report demonstrated the presence of HMW complexes containing MMP9, MMP2, MMP3, TIMP1, and TIMP2 in solid rat tissue (chorioallantoid membranes), but none has been reported in myocardium or skeletal muscles [11]. We have revealed that in porcine myocardium MMP9 forms 3 complexes together with MMP2, TIMP1, and NGAL. Whereas estimates of molecular weight of MMP9 monomer ranges from 80 kDa to 92 kDa, according to different authors [11, 36], NGAL monomer weights about 25 kDa (and spontaneously formed dimer ~50 kDa, [15]), TIMP1 monomer ~30 kDa [36], and MMP2 monomer ~72 kDa [38]. Therefore, the following constituents of HMW complexes can be suggested, 220 kDa complex: MMP2, MMP9, NGAL, and TIMP1; 170 kDa complex: MMP9, NGAL (dimer), and TIMP1; 130 kDa complex: MMP9, NGAL, and TIMP1. The 220 kDa complex appears to be stable in reducing and nondenaturing conditions, and labile in strong reducing and denaturing conditions suggesting that hydrophobic interactions may play a role in the maintenance of its stability. In contrast, the other 130 and 170 kDa complexes were destabilized even in reducing and nondenaturing conditions suggesting the involvement of disulphide bonds in their formation. Also the other authors have confirmed that the integrity of heterogeneous MMP9 complexes results from the presence of both disulfide bonds and hydrophobic interactions [39, 40]. To the best of our knowledge, our study is the first report on the presence of HMW heterogeneous complexes containing MMP9, MMP2, NGAL, and TIMP1 in both failing and normal myocardium.

It has been shown in *in vitro* studies that the formation of MMP9-NGAL complex can attenuate the autodegradation of MMP9 [9]. Also, HMW complexes containing MMP9 have been postulated to serve as a reservoir of active MMP9 protecting it from the rapid degradation when present in biological fluids [13, 15]. The evidence on the analogous role of such complexes in solid tissues is missing. We were able to demonstrate for the first time that HMW complexes containing MMP9 and MMP2 are present in myocardium (but not in skeletal muscles) and are proteolytically inactive but can spontaneously release active MMP9 and MMP2. This observation indicates that the aforementioned HMW complexes may serve as the tissue reservoir of active MMP9, which seems to be unique for myocardial tissue.

Taking into consideration the firmly established role of MMP9 and MMP2 in myocardial remodeling and HF development [4], one might expect differences in the mRNA and protein expression along with proteolytic activity of MMP9 (and also MMP2) between animals with and without systolic HF. In our study, there were no differences in the expression of mRNA and protein of MMP9, MMP2, and TIMP1 in either LV myocardium or skeletal muscles between diseased and healthy animals. These data suggest that the activity of these enzymes may be altered posttranslationally in heart failure and is not dependent on the expression of the mRNA or protein. The only observed difference was the gradual decline in the expression of both mRNA NGAL and the protein amount of NGAL in LV myocardium (but not in skeletal muscles) in male pigs along with the progression of systolic HF. The decline in the mRNA NGAL expression in porcine LV myocardium was accompanied by LV dilatation, the augmented diastolic dysfunction, and the increased PRA activity. So far, the NGAL expression in myocardium has been studied in rodent models of myocardial infarction (MI) and hypoxia [33, 41], where the authors revealed the augmented myocardial expression of rodent NGAL homologue (24p3) in animals with heart dysfunction. Moreover, Aigner et al. [42] showed that an increase in murine myocardial NGAL seen after ischemia and reperfusion is associated with the presence of polymononuclear cells infiltrating myocardial tissue, and NGAL may play a role of an acute phase protein. The evidence on myocardial NGAL expression is scarce, but based on the aforementioned studies and our results, it may be presumed that the pattern on NGAL myocardial expression differs between ischemic and hypoxic myocardium [33, 41] and failing myocardium in animals with nonischaemic HF (our data).

In conclusion, HMW complexes without proteolytic activity containing MMP9, MMP2, TIMP1, and NGAL are present in healthy and failing porcine LV myocardium, but not in skeletal muscles. HMW complexes spontaneously become the source of *in vitro* proteolytically active MMP9 and MMP2. We should acknowledge that we have not investigated the links between the presence of these complexes in LV myocardium and the magnitude of myocardial remodeling. Further studies are warranted to delineate this process. The presence of MMP in complexes in myocardium should be taken into consideration when designing pharmaceutical interventions inhibiting the MMP system in order to combat the pathological myocardial remodeling.

## Figures and Tables

**Table 1:** Oligonucleotide primers used in RT-PCR experiments.

Gene	Primers	Sequence 5′-3′	Genbank accession no.
MMP9	sMMP9 f	CCACAGGCCCTCCTTCAG	NM001038004
	sMMP9 r	TGAACAGCAGCACCTTACC	
MMP2	sMMP2 f	TACACCTATACCAAGAACTTCCG	NM214192
	sMMP2 r	TGTCCGCCAGATGAACCG	
NGAL	sNGAL f	TTAAGAAATACTCTGGATTGC	AK240091
	sNGAL r	TACTCTTGGTTGTTGGAAAC	
TIMP1	sTIMP1 f	AGCCAGGAGTTTCTCATAGC	NM213857
	sTIMP1 r	TCACAGCCAGCAGCATAG	
GAPDH	GAPDHS	TCACTGCCACCCAGAAGA	ABO38240
	GAPDHAS	TACCAGGAAATGAGCTTGAC	

MMP9: matrix metalloproteinase type 9; MMP2: matrix metalloproteinase type 2; NGAL: neutrophil gelatinase-associated lipocalin; TIMP1: tissue inhibitor of metalloproteinases type 1; GAPDH: glyceraldehyde-3-phosphate dehydrogenase.

**Table 2:** Echocardiography parameters reflecting the structure and functioning of left ventricle and the measures of neurohormonal activation in sham-operated male pigs (controls) and right-ventricle-paced male pigs with induced heart failure.

Variables, units	Controls (*n* = 5)	Mild HF (*n* = 7)	Moderate HF (*n* = 8)	Severe HF (*n* = 5)	Spearman correlatory rank coefficients *R* with *P *	
					For all animals	Only for animals with HF
Echocardiography parameters						
LVEF, %	52 ± 4	42 ± 5	28 ± 5	20 ± 3	*R* = −0.72	*R* = −0.61
					*P* < 0.001	*P* = 0.004
LVFS, %	25 ± 3	22 ± 7	14 ± 7	10 ± 4	*R* = −0.74	*R* = −0.66
					*P* < 0.001	*P* = 0.001
LVESV, mL	93 ± 15	134 ± 32	213 ± 36	223 ± 41	*R* = 0.84	*R* = 0.72
					*P* < 0.001	*P* < 0.001
LVEDV, mL	192 ± 24	241 ± 36	284 ± 41	277 ± 32	*R* = 0.73	*R* = 0.49
					*P* < 0.001	*P* = 0.03
SV, mL	94 ± 8	107 ± 14	88 ± 20	55 ± 11	*R* = −0.38	*R* = −0.46
					*P* = 0.06	*P* = 0.04
LVPW thickening, %	49 ± 3	54 ± 4	45 ± 10	15 ± 7	*R* = −0.59	*R* = −0.71
					*P* = 0.02	*P* < 0.001
LA/Ao	1.33 ± 0.06	1.91 ± 0.10	1.9 ± 0.13	2.57 ± 0.14	*R* = 0.73	*R* = 0.56
					*P* < 0.001	*P* = 0.01
Em, m/s	0.19 ± 0.02	0.18 ± 0.03	0.15 ± 0.04	0.15 ± 0.04	*R* = −0.42	*R* = −0.27
					*P* = 0.04	*P* = 0.25
Am, m/s	0.11 ± 0.03	0.08 ± 0.01	0.07 ± 0.01	0.07 ± 0.01	*R* = −0.31	*R* = −0.09
					*P* = 0.12	*P* = 0.68
Measures of neurohormonal activation						
BNP, ng/mL	0.25 ± 0.25	0.29 ± 0.19	0.65 ± 0.46	0.80 ± 0.30	*R* = 0.61	*R* = 0.58
					*P* = 0.001	*P* = 0.009
PRA, ng/mL/h	0.3 ± 0.2	1.0 ± 0.4	2.0 ± 0.7	4.1 ± 1.9	*R* = 0.66	*R* = 0.48
					*P* = 0.001	*P* = 0.07

HF: heart failure; LVEF: left ventricular ejection fraction; LVFS: left ventricular fractional shortening; LVESV: left ventricular end-systolic volume; LVEDV: left ventricular end-diastolic volume; SV: stroke volume; LVPW: left ventricular posterior wall; LA/Ao: left atrial/aorta ratio; Em: early diastolic velocity ratio; Am: late diastolic velocity ratio; BNP: B-type natriuretic peptide; PRA: plasma renin activity. All echocardiography measures were performed directly before an euthanasia. Data are presented as arithmetical means ± standard errors of a mean.
